# Supplementary material for: Exploiting a cognitive bias promotes cooperation in social dilemma experiments
Source: Nat Commun. 2018 Jul 27;9:2954. doi: 10.1038/s41467-018-05259-5 (PMC6063929; doi:10.1038/s41467-018-05259-5)
Supplement: Supplementary file 1 — Supplementary Information File [file 41467_2018_5259_MOESM1_ESM.pdf]

—Supplementary Information—

Exploiting a cognitive bias promotes cooperation in social dilemma experiments

Wang et al.

Supplementary Methods

Experimental Protocol and Volunteer Recruitment. The final version of the experimental protocol for the present study was prepared in September 2015. To ensure a degree of comparability with previous social dilemma experiments, we minimally modified an existing experimental protocol[1–3]. Specifically, our protocol envisioned two main treatments. A *control* treatment consisted of volunteers playing a repeated Prisoner's Dilemma (rPD) game in which the only two actions available where cooperation C and defection D. A decoy treatment introduced the third action which we interpreted as reward R because choosing this action meant giving more than in the case of C, but the opponent would also receive more (Equation 1). Two additional treatments were organised to test whether volunteers correctly perceived the value of R relative to C. In all treatments, volunteers would engage in multiple encounters comprised of multiple rounds. The term *encounter* referred to an rPD game between the same pair of opponents, while the term *round* referred to a single repetition of that game. The described protocol was implemented over the course of 11 experimental sessions from October 2015 to April 2016 (Supplementary Table 1). These sessions took place at the computer lab of Yunnan University of Finance and Economics, Kunming city, Yunnan province, south-western China. The lab was equipped with around 100 computer cubicles, designed to minimise chatter between participants during the experiment.

We recruited 328 undergraduate volunteers majoring in mathematics and natural sciences (M&NS; 177 people) or humanities and social sciences (H&SS; 151 people). These volunteers came from two universities located in Kunming city: Yunnan University of Finance and Economics and Yunnan University. Details on the protocol were not revealed to volunteers during recruitment.

Instead, volunteers were just required to show up at a designated location on the appointed date to participate in an experimental session. All sessions were carried out on dates that avoided a possible conflict with the scheduled lectures (Supplementary Table 1).

The number of encounters in a single experimental session was determined by two rules. First, volunteers were paired in a random order, but in a manner that avoided having the same pair meet over and over again. In a session with N volunteers, this rule would have limited the number of encounters to $N-1$ before pairs who have already played started to reappear. Second, each session was timed to last about 75 minutes to maximise the amount of data obtained before volunteers lost their concentration. Within this time period, $\approx$20 encounters could take place, as reflected in the actual numbers of realised encounters (Supplementary Table 1).

The number of rounds to be played in a single encounter was determined randomly. The first round was certain, but the probability of terminating the encounter after any one round was set to 25%. To ensure that nobody sits idle, one random draw was made for all pairs, meaning that every volunteer played the same number of rounds. According to the mentioned termination probability, the expected number of rounds in an encounter was 4 with a standard deviation of $\approx$3.5. Given this expected number of rounds and the approximate number of encounters in an experimental session, the total number of rounds per session was $\approx 4 \cdot 20 = 80$ (Supplementary Table 1).

To avoid contaminating the results as the end of a session approached, the exact number of encounters was kept undisclosed. However, the probability of playing another round of an encounter was made known. Volunteers furthermore knew about random rematching after the completion of every encounter. Two supervisors were present at all times to answer questions that

volunteers may have had about the experiment or the experimental protocol. These supervisors also acted to prevent chatter.

Volunteers were recruited mostly from freshman and sophomore years because in this way we could recruit a sufficient number of individuals who have never participated in social dilemma experiments. This choice led to a young average age of volunteers ($\approx$19–20 years) and a rather low standard deviation ($\approx$0.90 years). Ideally, we would like to keep the ratio of women to men at around 1:1, but this was not exactly possible for the same reason as above—we needed a relatively large number of volunteers without any prior experience with social dilemma experiments. We analysed the role of gender as a confounding factor to understand the potential bias in the results that an uneven sex ratio may have caused (Supplementary Note 3).

Experimental Platform and Interface. We developed an experimental platform using the z-Tree software package[4] to allow volunteers to interact with one another. This platform contained two main interfaces with the following key elements (Supplementary Figure 1): (i) the number of the current round and the current encounter in the top left box, e.g., 3/13 indicated the third round of the thirteenth encounter; (ii) the remaining time to make an action choice in the top right box; and (iii) a brief description in the middle box of the three possible action choices denoted by “1”, “2”, and “3” instead of C, D, and R to avoid framing effects. Action choices had to be made within 30 seconds by entering a number corresponding to the desired action (1, 2, or 3) into the light blue rectangle (Supplementary Figure 1**A**). All selections had to be confirmed by clicking the “OK” button on the lower right side of the interface. An automatic selection would be made for those participants who could not make a decision within the allotted time. However, such an automatic

selection by the system has not been triggered.

The platform moved to the second interface screen (Supplementary Figure 1**B**) as soon as all volunteers finished inputting their action choices. The second interface was designed to allow reviewing the outcome of the current round. Only the bottom box was different from the first interface. The newly displayed information included: (i) own and opponent's action choices in the current round; (ii) own and opponent's payoff in the current round; and (iii) the updated total payoff. Volunteers proceeded to the next round or to a new encounter by clicking the "continue" button in the lower right corner or automatically after 30 seconds.

Instructions. Here we provide a complete translation of the instructions displayed to volunteers prior to engaging in an experimental session (Supplementary Figure 2).

$\star$ $\star$ $\star$ Screen 1 $\star$ $\star$ $\star$

Welcome to our game experiment on human behaviour!!!

Please read the following instructions carefully. If you have any questions, please raise your hand and ask the staff without hesitation. Communication during the experiment is forbidden. The experiment is completely anonymous. To ensure anonymity, you will be assigned a random ID number at the beginning of the game, and this will be your only identifying tag until the game ends.

Game rules:

1. Encounters: The game is composed of an unknown number of encounters, each of which

consists of several rounds. For each encounter, you will be randomly matched with another person, who will act as your opponent for the duration of this encounter. You will only know your opponent's ID number to keep the game strictly anonymous. Furthermore, the number of rounds in an encounter is also unknown. What is known is the termination probability of 25% that the encounter will finish after any particular round. When the encounter finishes, everyone will be randomly re-matched to play against another opponent.

Note: Random re-matching is performed by the computer system to secure complete anonymity. The system also determines the number of rounds in each encounter.

2. Payout: Before the game, everyone will receive a fixed amount of ¥15 as a show-up fee, and 50 units of initial score. Based on your final score, you will receive additional monetary payout as follows:

   (1) If you accumulate a positive number of units, your total monetary payout will be given by formula: score$\times$0.2 + show-up fee.

   (2) If you accumulate zero or a negative number of units, you will only get the show-up fee.

`* * * Screen 2 * * *`

3. Action selection: In each round, there are three actions available to both you and your opponent: action 1, action 2, and action 3. The effect of these actions can be summarised as follows:

	You	Opponent
Action 1	-1	$+2$
Action 2	$+1$	-1
Action 3	-2	$+3$

Notes:

- If you choose action 1, you give up 1 unit, but your opponent gets 2 units.
- If you choose action 2, you get 1 unit, but your opponent loses 1 unit.
- If you choose action 3, you give up 2 units, but your opponent gets 3 units.
- Conversion at payout is 1 unit = ¥0.2.

4. Interface information: Throughout the game you will consult two main interfaces: the *action-selection interface* and the *results interface*.

   (1) The action-selection interface will give you and your opponent 30 s to select one action. After you finish your selection, please click the "OK" button in the bottom right corner. If you do not make a selection within the allotted time, the system will randomly choose one action for you, and jump to the next interface screen automatically.

   (2) The result interface will give you and your opponent another 30 s to examine the consequences of the selected actions. The displayed information includes: a) your opponent's ID, b) your and the opponent's payoffs in the current round, c) your and the opponent's action choices in the current round, and d) your accumulated score. After consulting all this information, please click the "continue" button in the bottom right corner. If you fail to click within the allotted time, system will jump to the next interface screen automatically.

$\star$ $\star$ $\star$ Screen 3 $\star$ $\star$ $\star$

Examples:

1. If you and your opponent choose action 1, both of you will receive 1 unit of score ($-1+2=+1$, i.e., your action 1 gives up 1 unit, but the opponent's action 1 gives you 2 units, for a

total of $+1$).

2. If you choose action 1 and your opponent chooses action 3, you will receive 2 units of score ($-1 + 3 = +2$, i.e., your action 1 gives up 1 unit, but the opponent's action 3 gives you 3 units, for a total of $+2$). Meanwhile, your opponent will receive 0 units ($+2 - 2 = 0$, i.e., your action 1 gives the opponent 2 units, but their action 3 loses them 2 units, for a total of 0).

3. If you choose action 3 and your opponent chooses action 2, you will receive negative 3 units of score ($-2 - 1 = -3$, i.e., your action 3 gives up 2 units, and your opponent's action 2 loses you another 1 unit, for a total of -3). Meanwhile, your opponent will receive 4 units ($+1 + 3 = +4$, your action 3 gives the opponent 3 units, and their action 2 wins them additional 1 unit, for a total of $+4$).

Pre-game Test. After reading the instructions, we asked volunteers to take a two-question pre-game test (Supplementary Figure 3). This test was a precautionary measure to detect those individuals who lacked the most basic understanding of the rPD game rules. Both question had to be answered correctly to participate in an experimental session. Only two volunteers failed the test, after which they were paid the show-up fee and sent home before they could play the rPD game.

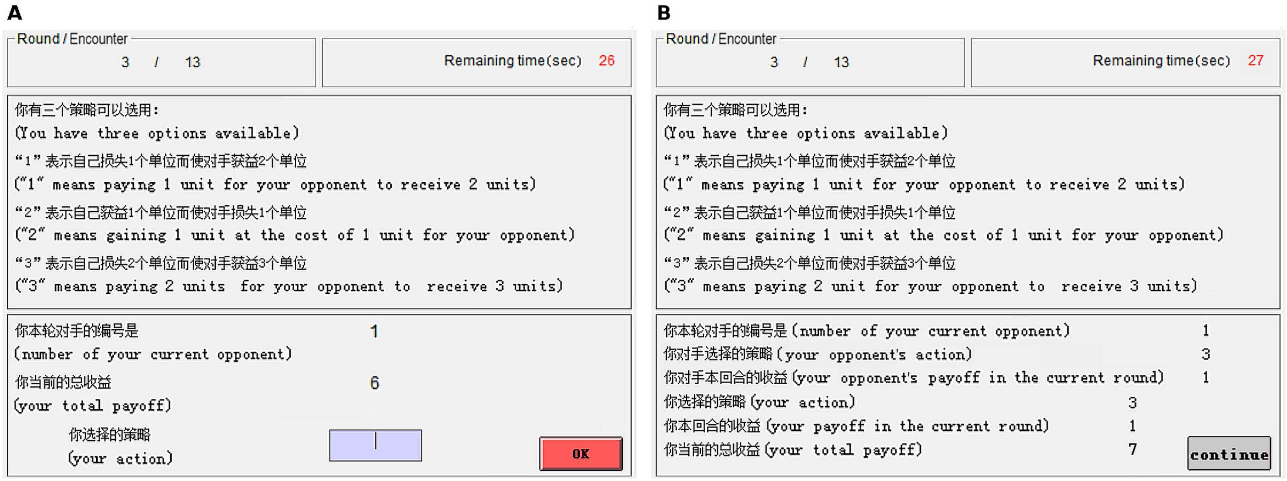

Supplementary Figure 1: Computer interface for playing rPD in the decoy treatment of the present social dilemma experiment. A Screen for selecting an action in a single round of an encounter. Volunteers were given 30 seconds to express their choice. Actions “1”, “2”, and “3” were interpreted as *cooperation*, *defection*, and *reward*, respectively. However, to avoid undesired framing, these interpretations remained undisclosed. **B** Screen for inspecting the outcome of a single game round. Volunteers were given additional 30 seconds to inspect the consequences of their current action, as well as the cumulative total payoff since the beginning of the experiment.

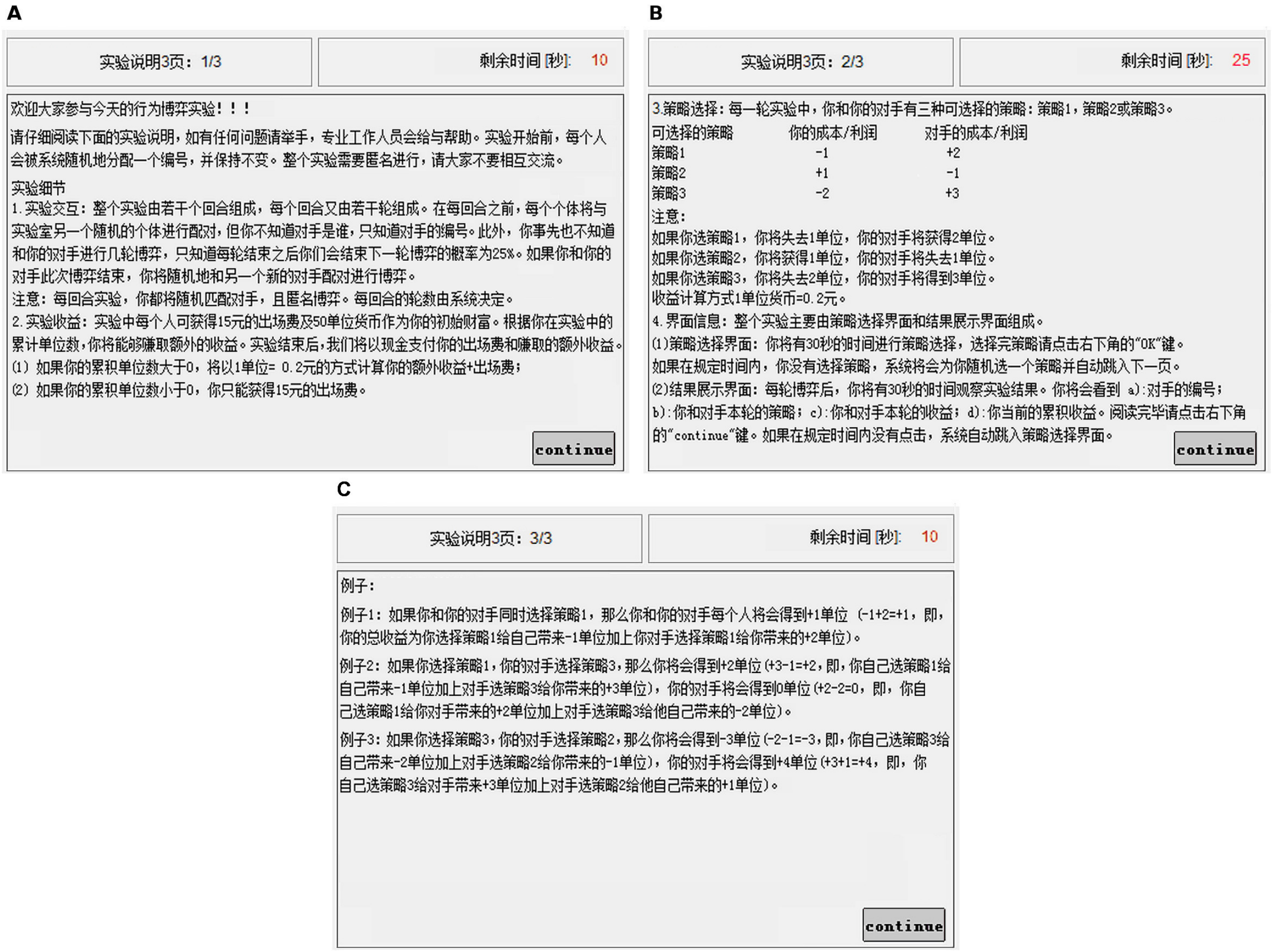

Supplementary Figure 2: **Instructions for playing rPD in the decoy treatment of the present social dilemma experiment.** **A**, **B** Screens with instructions that were presented to volunteers before an experimental session would start. **C** Instructions were accompanied with three simple examples explaining the effects of each of the three available actions. A full English translation of these screens is given in the text.

调查问卷

Pre-game test

A

在囚徒困境博弈中（如下收益矩阵），你和你的对手同时进行策略选择。假如，你当前只有一个对手，你现在的总收益为 25，你和你的对手分别选择策略“1”和“2”。那么这轮你获得的收益为________，这轮之后你的总收益为_________。

B

假如你现在有两个对手，你现在的总收益仍为 25，这一轮中你的策略为“2”，你两个对手的策略分别为“1”和“3”。那么这轮你获得的收益为______，这轮之后你的总收益为________。

收益矩阵

Payoff matrix

$$\begin{array}{c} \\ \text{策略 1} \\ \text{策略 2} \\ \text{策略 3} \end{array} \begin{array}{c} \text{你} \quad \text{对手} \\ \begin{pmatrix} -1 & +2 \\ +1 & -1 \\ -2 & +3 \end{pmatrix} \end{array}$$

Supplementary Figure 3: **Pre-game test to filter out volunteers without the basic understanding of the rPD game rules.** To participate in an experimental session, volunteers had to answer both questions correctly. Here follows an English translation of the test questions. **A**, In a Prisoner's Dilemma game (see the accompanying payoff matrix), players make action choices simultaneously. Assuming that you have one opponent and your current total payoff is 25, if you and your opponent respectively choose actions “1” and “2”, you earn ____ in the current round, and your total payoff becomes ____ after this round. **B**, Now you have two opponents at the same time. Your current total payoff is again 25. If you choose action “2”, and your two opponents respectively choose actions “1” and “3”, you earn ____ in the current round, and your total payoff becomes ____ after this round.

Supplementary Table 1: **Basic information on the experimental sessions.**

Treatment	Date	Encounters	Rounds	Volunteers	Mean (SD) age	% women
Decoy	17 Oct 2015	25	84	22	20.7 (0.88)	91
	04 Nov 2015	25	80	36	18.9 (0.92)	56
	05 Nov 2015	17	81	40	18.6 (1.43)	83
$\alpha = 4$	25 Oct 2016	31	81	36	20.5 (0.94)	72
	25 Oct 2016	23	80	40	19.7 (0.82)	73
	25 Oct 2016	16	85	42	18.6 (0.88)	67
$\alpha = 5$	07 Nov 2015	21	81	34	20.6 (1.60)	65
	17 Nov 2015	25	80	40	20.8 (0.98)	55
	17 Apr 2016	23	81	38	19.3 (0.93)	71

A total of 11 sessions was split between a control (detailed before[3]), a decoy, and two other treatments designed to test whether participants understood the value of reward R relative to cooperation C. Each session was characterised by the number of encounters, the number of rounds, attendance, the mean age of volunteers and its standard deviation, and the percentage of women.

Supplementary Note 1

The dilemma strength parameters[5], denoted D'_{g} and D'_{r}, quantify respectively how "lucrative" and how "safe" defection is relative to cooperation, thereby reducing dimensionality of 2×2 evolutionary games from four to two. More specifically, 2×2 evolutionary games are defined in terms of payoff matrices of the form

$$\begin{array}{c c} & \begin{matrix} C & D \end{matrix} \\ \begin{matrix} C \\ D \end{matrix} & \begin{pmatrix} R & S \\ T & P \end{pmatrix} \end{array}, \tag{1}$$

where R is called reward (not to be confused with action R), S is sucker's payoff, T is temptation, and P is punishment. Using these payoffs, we can define the dilemma strength parameters as

$$D'_{\mathrm{g}} = \frac{T - R}{R - P}, \tag{2a}$$

$$D'_{\mathrm{r}} = \frac{P - S}{R - P}. \tag{2b}$$

Supplementary Equation (2a) dictates that, as T increases relative to R, the positive value of D'_{g} becomes larger. This characterises a situation conducive to defection because temptation gradually trumps reward received for mutual cooperation. The normalisation factor, $R-P$, must be taken into account because both more generous reward and more stringent punishment dissuade defection, meaning that increasing $R - P$ acts in the opposite direction of increasing $T - R$. Accordingly, parameter D'_{g} quantifies the extent to which attempting defection is worthwhile. Similar reasoning applies to D'_{r} in Supplementary Equation (2b) because making S more negative relative to P signifies that punishment for mutual defection gradually pales in comparison to being exploited by a defector, which is again conducive to defection. The difference is that D'_{r} quantifies the extent

to which attempting defection is harmless. These interpretations provide an intuition as to why dilemma strength parameters $D_{\mathrm{g}}^{'}$ and $D_{\mathrm{r}}^{'}$ might carry the same information content as payoffs R, S, T, and P.

We start formalising the last statement by comparing payoff orderings in four famous variants of 2×2 evolutionary games: Prisoner's Dilemma (PD), Hawk-Dove (H-D), Stag Hunt (SH), and Harmony (H). In a PD game, payoff ordering is $T > R > P > S$ implying that $D_{\mathrm{g}}^{'} > 0$ and $D_{\mathrm{r}}^{'} > 0$. This payoff ordering is known to hamper cooperation, which is consistent with the above interpretation of the dilemma strength parameters whereby large positive values of $D_{\mathrm{g}}^{'}$ and $D_{\mathrm{r}}^{'}$ are conducive to defection. In an H-D game, emphasis is on the severity of punishment. The corresponding payoff ordering is $T > R > S > P$ implying that $D_{\mathrm{g}}^{'} > 0$, but $D_{\mathrm{r}}^{'} < 0$. This particular payoff ordering leads to coexistence of cooperators and defectors, which is consistent with the interpretation that a negative value of $D_{\mathrm{r}}^{'}$ signifies severe harm from mutual defection. In this case, it is better to be an exploited cooperator than caught in the act of mutual defection. Payoff ordering in an SH game is $R > T > P > S$ leading to $D_{\mathrm{g}}^{'} < 0$, but $D_{\mathrm{r}}^{'} > 0$. This arrangement has high enough reward to produce bi-stability. A population of players may end up cooperating or defecting depending on initial conditions. Finally, in an H game, payoff ordering $R > T > S > P$ implies $D_{\mathrm{g}}^{'} < 0$ and $D_{\mathrm{r}}^{'} < 0$. In this case, both reward for mutual cooperation and punishment for mutual defection are high enough to jointly discourage defection. A summary of these considerations is presented in Supplementary Figure 4.

The reason why the dilemma strength parameters reduce dimensionality of 2×2 evolutionary games is an invariance theorem[6] stating that if the elements, M_{ij}, of a given payoff matrix are

transformed using an affine transformation, $M'_{ij} = aM_{ij} + b$, the dynamics of selection remain unaltered. Therefore, the following equivalence holds

$$\begin{pmatrix} R & S \\ T & P \end{pmatrix} \Leftrightarrow \begin{pmatrix} 1 & \frac{S-P}{R-P} \\ \frac{T-P}{R-P} & 0 \end{pmatrix} = \begin{pmatrix} 1 & -D'_{\mathrm{r}} \\ 1+D'_{\mathrm{g}} & 0 \end{pmatrix}. \tag{3}$$

We further simplify our considerations by setting $\mathrm{DS} \equiv D'_{\mathrm{g}} = D'_{\mathrm{r}}$ in all instances.

Based on the payoffs defined in Equation 1 of the main text and the definition of dilemma strength in Supplementary Equation (2), our control treatment has $\mathrm{DS} = 2$. Pair (D, R) in the decoy treatment has $\mathrm{DS} = 3$, which decreases to $\mathrm{DS} = 1.5$ and $\mathrm{DS} = 1$ in the additional treatments with reward parameter $\alpha = 4$ and $\alpha = 5$, respectively. These dilemma strengths imply that initially reward R is an inferior choice to cooperation C, but this situation reverses in the additional treatments.

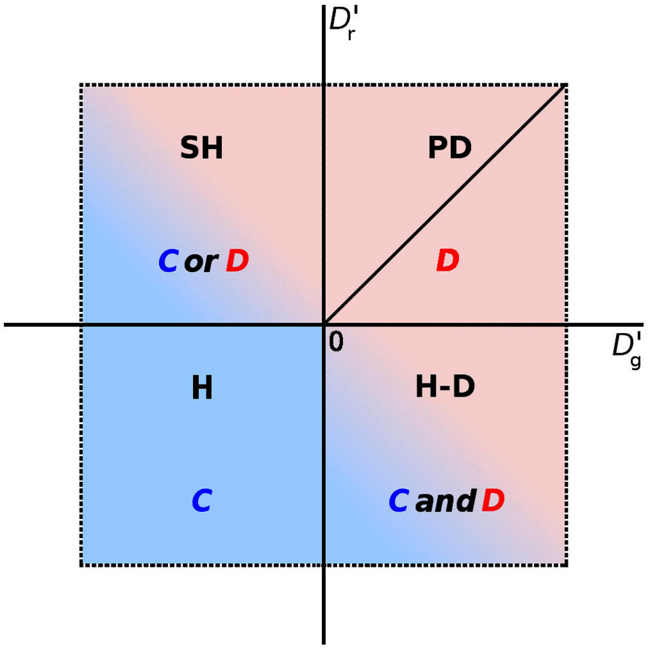

Supplementary Figure 4: **Dilemma strength determines the nature of 2×2 evolutionary games.** Positive $D_{\rm g}^{'}$ and $D_{\rm r}^{'}$ signify a Prisoner's Dilemma game conducive to defection. Keeping $D_{\rm g}^{'}$ positive while making $D_{\rm r}^{'}$ negative emphasises the severity of punishment for mutual defection in which case the game is Hawk-Dove and cooperators coexist with defectors. Making $D_{\rm g}^{'}$ negative while keeping $D_{\rm r}^{'}$ positive changes the game into Stag Hunt wherein bi-stability ensures that either cooperators or defectors eventually prevail. Finally, negative $D_{\rm g}^{'}$ and $D_{\rm r}^{'}$ characterise the Harmony game conducive to mutual cooperation. In the present experiment, we consider only positive and equal $D_{\rm g}^{'}$ and $D_{\rm r}^{'}$, thus remaining on the diagonal within the realm of the PD game.

Supplementary Note 2

In the present experiment, volunteers play a repeated Prisoner's Dilemma (rPD) game. Repetitions are an important aspect of the game which was not considered in the section on the dilemma strength parameters. To understand the effect of repetitions we assume that volunteers are either defectors or conditional (i.e., tit-for-tat) cooperators[7]. This assumption implies that in the control treatment, the rPD game's outcome is completely determined by payoff matrix[7]

$$\begin{pmatrix} \frac{R}{q} & S+\frac{1-q}{q}P \\ T+\frac{1-q}{q}P & \frac{P}{q} \end{pmatrix} = \begin{pmatrix} 4 & -2 \\ 3 & 0 \end{pmatrix}, \tag{4}$$

where q is the termination probability set to 25% throughout the experiment. We see that repetitions make the *expected* reward for mutual cooperation greater than the expected temptation ($4 > 3$), thus—in a probabilistic sense—transforming the original PD game into a bi-stable SH game. Conditional cooperators may therefore prevail given that their initial fraction within the population is sufficient. The potential prevalence of conditional cooperators is seen using the replicator dynamics. Let us denote the fraction of conditional cooperators in the population with x_C. Whether x_C increases or decreases depends on the sign of derivative

$$\frac{dx_C}{dt} = x_C(1-x_C)(\pi_C - \pi_D), \text{ where} \tag{5a}$$

$$\pi_C = x_C\frac{R}{q} + (1-x_C)\left(S + \frac{1-q}{q}P\right), \text{ and} \tag{5b}$$

$$\pi_D = x_C\left(T + \frac{1-q}{q}P\right) + (1-x_C)\frac{P}{q}. \tag{5c}$$

Quantities π_C and π_D are the expected payoffs of conditional cooperators and defectors, respectively, which in the control treatment amount to $\pi_C = 2(3x_C - 1)$ and $\pi_D = 3x_C$ (Supplementary Equation 4). There is one non-trivial equilibrium at $x_C^* = \frac{2}{3}$. If the initial fraction of conditional

cooperators is $x_C < \frac{2}{3}$, then $\frac{dx_C}{dt} < 0$ and defectors dominate. If the opposite condition is satisfied, $x_C > \frac{2}{3}$, then $\frac{dx_C}{dt} > 0$ and conditional cooperators dominate (Supplementary Figure 5).

In the decoy treatment, as well as the additional treatments with $\alpha = 4$ and $\alpha = 5$, reward R (action, not payoff) is another form of cooperation. R is more costly than C, but also brings more benefit to an opponent. Conditional cooperators should, therefore, choose between C and R based on which of these two options improves their payoff. Once the right choice is made, the other option should be disregarded. This leads us to investigate whether R fares better against D than C, depending on the value of reward parameter α. Following the same steps as above yields $\pi_D = (4\alpha - 5)x_R - 3$ and $\pi_R = (\alpha + 1)x_R$, where x_R plays the role of x_C. For $\alpha = 3$, we do not find a non-trivial equilibrium, implying that $\frac{dx_R}{dt} < 0$ for all $0 \leq x_R < 1$. For $\alpha = 4$, the non-trivial equilibrium is $x_R^* = \frac{1}{2}$, meaning that $\frac{dx_R}{dt} < 0$ ($\frac{dx_R}{dt} > 0$) if $x_R < \frac{1}{2}$ ($x_R > \frac{1}{2}$). Finally, for $\alpha = 5$, the non-trivial equilibrium is $x_R^* = \frac{1}{3}$, meaning that $\frac{dx_R}{dt} < 0$ ($\frac{dx_R}{dt} > 0$) if $x_R < \frac{1}{3}$ ($x_R > \frac{1}{3}$). A visual summary of these considerations is shown in Supplementary Figure 5.

It remains unclear whether the dilemma strength parameters introduced in the preceding section are still valid predictors of outcome in an rPD game. The answer to this question is positive[5]. If $D_{\mathrm{g}}^{'} < \frac{1-q}{q}$, cooperation is an evolutionarily stable strategy (ESS). If in addition $D_{\mathrm{r}}^{'} > 0$, defection is also an ESS. An interior equilibrium is given by $x_C^* = \frac{qD_{\mathrm{r}}^{'}}{q(D_{\mathrm{r}}^{'} - D_{\mathrm{g}}^{'}) + 1 - q}$. If the initial fraction of conditional cooperators is below (above) this equilibrium value, evolutionary dynamics leads to the prevalence of defectors (conditional cooperators). For the (C, D) pair we obtain $x_C^* = \frac{2}{3}$, which corresponds to the situation in Supplementary Figure 5. The dilemma strength parameters are therefore a valid indicator of which dilemma pair is preferable, (C, D) or (D, R).

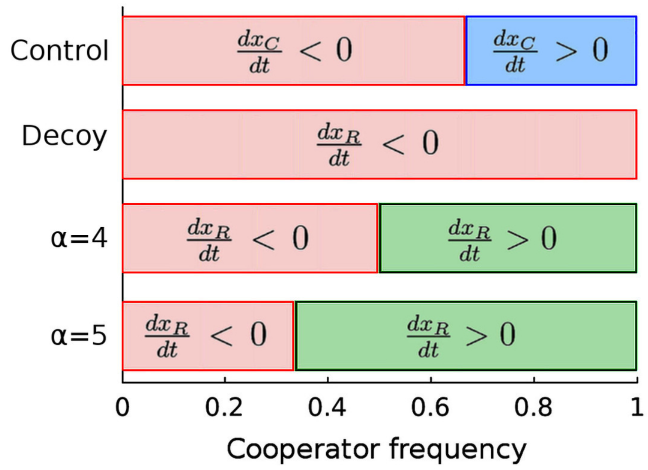

Supplementary Figure 5: **Evolutionary dynamics of conditional cooperators in 2×2 rPD games.** Pair (C, D) is shown in the first row. Pair (D, R) is shown in the remaining three rows, depending on the value of reward parameter α. For $\alpha = 3$, R should be disregarded in favour of C because the latter option offers a better payoff and thereby some resistance against defectors. For $\alpha = 4, 5$, it is R that offers more protection against defectors, thus emerging as a preferable option to C.

Supplementary Note 3

We investigated the role of gender and academic background as confounding factors that may affect the behaviour of volunteers in social dilemma experiments. To this end, we summarised how volunteers played the repeated Prisoner's Dilemma (rPD) game depending on treatment, on the one hand, and gender (Supplementary Table 2) or academic background (Supplementary Table 3), on the other hand. This summary has the form of three-way contingency tables, suggesting that the relationships between action choices and confounding factors across treatments could be disentangled by means of log-linear models. We used the Akaike's Information Criterion (AIC) to identify the best fitting model. Furthermore, to provide intuition behind a particular model selection, we appended the contingency tables to show the frequencies of each action.

When examining gender as a confounding factor (Supplementary Table 2), the frequencies suggest that, for a fixed gender, action choices differ between treatments, but within a given treatment, women and men seem to behave similarly. This would further suggest that a joint-independence model with treatment$\times$action interaction might explain a large part of the variation in the data. Indeed, we find that the joint-independence model has AIC=528.9, while the simplest mutual-independence model without any interaction terms has AIC=9515. For a fixed action, however, there is a significant variation in the behaviour of genders across treatments, which leads to AIC=273.2 for a conditional-independence model with additional interaction term treatment$\times$gender. In the end, even the performance of the conditional-independence model is beaten by the most complex saturating model which yields AIC=194.2. Despite our intuition that interaction treatment$\times$action may describe the data in sufficient detail, we have to conclude oth-

erwise. Women are somewhat more likely to defect in the decoy treatment, while the opposite is true in the additional treatment with $\alpha = 5$. Both genders seem to recognise the value of R relative to C correctly. Overall, evidence suggests that action choices are significantly affected by gender across treatments despite the apparent similarities between genders.

Looking at academic background as a confounding factor (Supplementary Table 3), we find once again that the best fitting model is the saturating model (AIC=193.7), thus revealing that action choices are significantly affected by academic background across treatments. Unlike in the case of gender, this result is intuitively appealing because the frequencies in Table 3 show no discernible pattern when comparing (i) action choices between academic backgrounds within a given treatment, (ii) action choices across treatments for a fixed academic background, (iii) academic backgrounds across treatments for a fixed action choice, and (iv) the relationship between action choices and academic backgrounds across treatments. Nonetheless, we can make two interesting observations based on the values of these frequencies. First, in the decoy treatment, mathematics and natural sciences (M&NS) students tend to defect much less than their colleagues from humanities and social sciences (H&SS). Similar behaviour is present in the additional treatment with $\alpha = 5$, but not with $\alpha = 4$, perhaps due to some confusion about the roles of R and C. Speaking of such confusion, M&NS students are much better than H&SS students in sensing the value of R relative to C. Even with $\alpha = 4$, when the distinction between R and C is the least clear, M&NS students are more than $\frac{0.518}{0.167} \approx 3$ times as likely to choose R over C, while H&SS students seem to value these two actions approximately equally. With $\alpha = 5$, M&NS students are $\frac{0.776}{0.044} \approx 17.5$ times more likely to choose R over C, while for H&SS students the same ratio is only $\frac{0.470}{0.186} \approx 2.5$. Overall, academic background exerts a significant influence on students' behaviour.

Supplementary Table 2: **Contingency table for gender as a confounding factor.**

Treatment	Gender	Action		
		C	D	R
Decoy	Female	3230 (0.543)	2379 (0.400)	344 (0.058)
	Male	1229 (0.610)	692 (0.343)	94 (0.047)
$\alpha = 4$	Female	1544 (0.227)	2020 (0.297)	3242 (0.476)
	Male	516 (0.179)	860 (0.299)	1504 (0.522)
$\alpha = 5$	Female	516 (0.090)	1256 (0.219)	3957 (0.691)
	Male	327 (0.099)	882 (0.267)	2094 (0.634)

The table shows the number of times each available action was chosen depending on treatment and gender. The corresponding frequencies are shown in brackets. These frequencies are changing across treatments as the roles of cooperation C and reward R switch places. The differences between genders are not so obvious. Nevertheless, the best fitting log-linear model is the saturating model, thus indicating that gender affects behaviour in a significant manner. Women are somewhat more willing to defect in the decoy treatment, but this is reversed in the additional treatment with $\alpha = 5$. Both genders seem to value R relative to C in a similar fashion.

Supplementary Table 3: **Contingency table for academic background as a confounding factor.**

Treatment	Major	Action		
		C	D	R
Decoy	M&NS	1488 (0.771)	280 (0.145)	161 (0.083)
	H&SS	2971 (0.492)	2791 (0.462)	277 (0.046)
$\alpha = 4$	M&NS	1133 (0.167)	2131 (0.315)	3506 (0.518)
	H&SS	927 (0.318)	749 (0.257)	1240 (0.425)
$\alpha = 5$	M&NS	262 (0.044)	1065 (0.180)	4585 (0.776)
	H&SS	581 (0.186)	1073 (0.344)	1466 (0.470)

The table shows the number of times each available action was chosen depending on treatment and academic background. The corresponding frequencies are shown in brackets. These frequencies reveal considerable changes both across treatments and academic backgrounds. The former is expected due to reward R replacing cooperation C as the better option with the gradual increase of the reward parameter from $\alpha = 3$ to $\alpha = 5$. Reasons for the latter change are much less obvious, but one pattern that does emerge is that humanities and social sciences (H&SS) students are more likely to defect in treatments in which there is a clear difference between R and C (decoy and $\alpha = 5$). Mathematics and natural science (M&NS) students seem to be much better at assessing the value of R relative to C, which is seen particularly well in the most "confusing" treatment with $\alpha = 4$.

Supplementary Note 4

To test the noisy tit-for-tat play, we recreated the experiment in the form of simple numerical simulations in which agents make their choices based solely on the immediate preceding action of their opponents. We pooled the response probabilities of volunteers recorded in the decoy treatment (Figure 3) and randomly assigned those probabilities to the simulated agents. Each agent was thus given four triplets of probabilities; the first triplet consisted of the probabilities to respond to a cooperative opponent with (i) cooperation C, (ii) defection D, or (iii) reward R. Similar triplets were used to determine responses to an opponent's choice of D and R. Finally, one additional triplet was needed for the first round of an encounter to get the game started. We conducted 100 simulations, each with 100 agents who engaged in 99 encounters. This number of encounters is considerably larger than in a typical experimental session with $\approx$20 encounters, but we wanted to ensure that the results would be stable even in longer experimental sessions.

The simulation results show similar qualitative and quantitative patterns to the live experiment (Supplementary Figure 6). A typical simulation run exhibits somewhat more scatter than the experimental data (Supplementary Figure 6**A–C**), but this is attributable to the larger number of encounters in the simulations. Otherwise, the simulated average payoff per round as well as the correlation between payoff per round and the frequencies of cooperation C, defection D, and reward R match the experimental data very well (Supplementary Figure 6**D–F**). Our success in generating patterns similar to the observed ones by means of simple simulations based on the responses to the opponent's immediate preceding action provides a strong support for noisy tit-for-tat as the dominant mode of behaviour in rPD games.

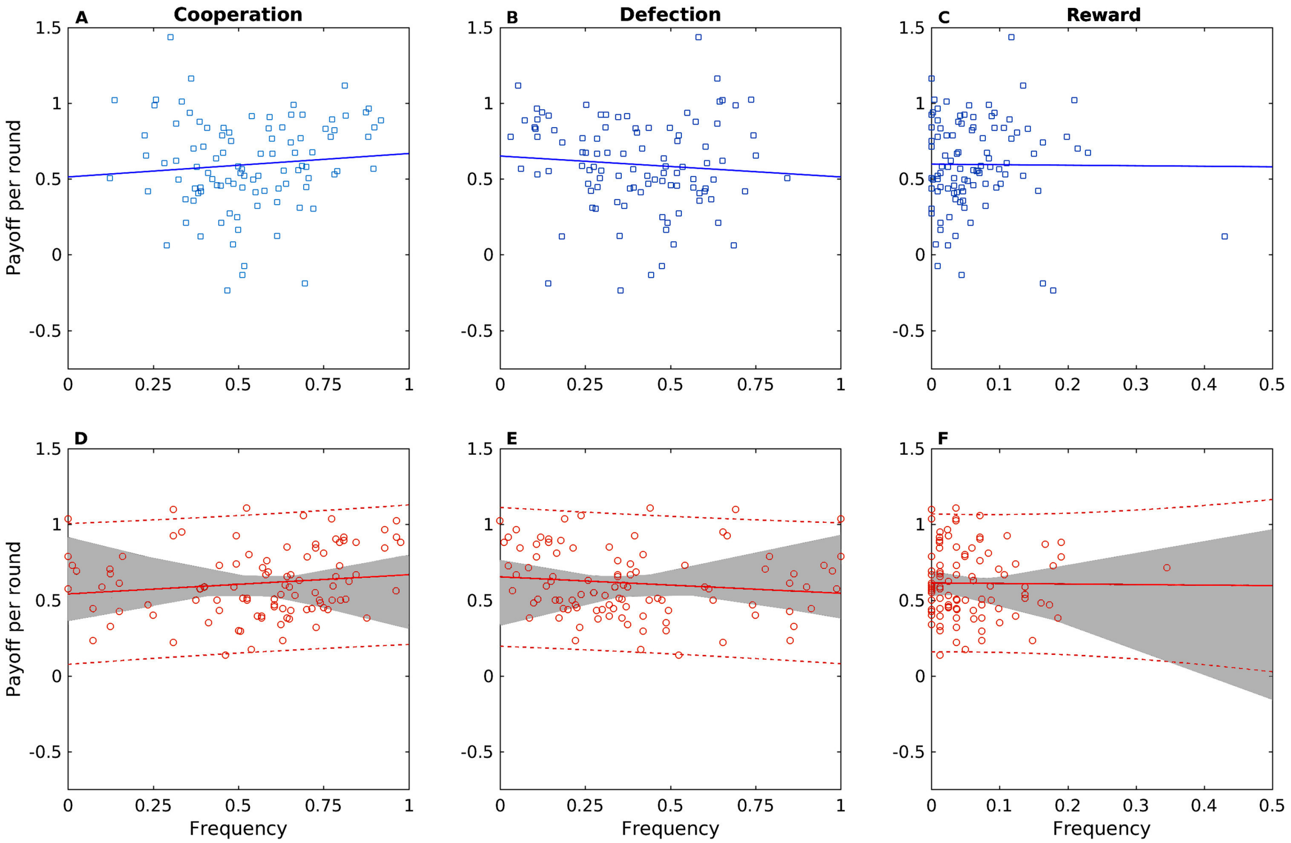

Supplementary Figure 6: **Computer simulations support noisy tit-for-tat as a major determinant of experimental outcomes.** We recreated the experiment in the form of simple computer simulations that take into account only how volunteers responded to an immediate preceding action of their opponents (see the distributions in Figure 3). **A–C**, The results of a typical simulation run with 100 players engaging in 99 encounters. These results exhibit a remarkable resemblance to the results of the live experiment (cf., panels **D–F**). Somewhat larger scatter in the simulations is explainable by the relatively large number of encounters compared to the experimental sessions (99 vs. ≈20, respectively). The reason for having a larger number of encounters in the simulations is to confirm that the results would not deviate far from the recorded ones even in considerably extended experimental sessions. **D–F**, Regression lines for the decoy treatment from Figure 5 superimposed on the 99% confidence interval for this same regression, but generated in the simulations (grey areas). A good agreement between the simulations and the results from the decoy treatment suggests that action choices of volunteers are to a large degree governed by actions that immediately preceded the moment of choosing. Furthermore, because cooperation is largely met with cooperation, defection with defection, and reward with more reward or cooperation (Figure 3), we conclude that tit-for-tat (i.e., conditional cooperation) is the predominant strategy determining the outcome of experimental rPD games.

Supplementary Note 5

Despite strong evidence that volunteers rely heavily on noisy tit-for-tat, we also examined how their cooperativeness develops within an encounter. This is an interesting question in view of Ref. [1], in which players become less cooperative with the increasing number of rounds, Ref. [2], in which the results are mixed, and Ref. [3], in which a similar decrease in cooperation is entirely absent.

In the present study, the cooperation frequency decreases significantly with each round played because volunteers keep gradually replacing cooperation with defection (Supplementary Figure 7). This replacement is seen in both treatments, but it is much faster in the decoy treatment. Reward has no effect in this context, as evidenced by the constant and low reward frequency within encounters. Accordingly, "noise" in noisy tit-for-tat does not cancel out completely, but rather exhibits a second-order effect of being biased towards defection. The magnitude of this bias is considerable and can be assessed by taking into account that the average cooperation frequency in the first round alone is 74.7%, but the average throughout the experiment is 54.7% (Figure 1).

From these results, we conclude that the decoy effect wears off throughout the course of a single encounter. Nonetheless, at the beginning of the next encounter, the decoy's effectiveness is renewed again, thus ultimately maintaining much higher cooperativeness than in the control treatment.

A

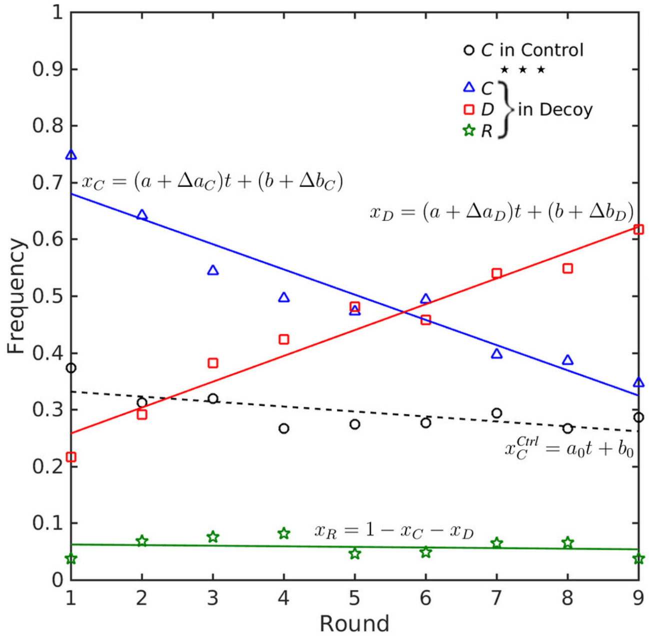

B

	Coefficient		Estimate	Significance
Control	Intercept	b_0	0.340	$p<10^{-11}$
	Slope	a_0	-0.009	$p=0.036$
Decoy	Intercept	b	0.468	$p<10^{-12}$
	Cooperation	Δb_C	0.256	$p<10^{-8}$
	Defection	Δb_D	-0.256	$p<10^{-8}$
	Slope	a	5.250×10^{-4}	$p=0.880$
	Cooperation	Δa_C	-0.045	$p<10^{-8}$
	Defection	Δa_D	0.045	$p<10^{-8}$

Supplementary Figure 7: **Within-encounter cooperativeness decreases with the increasing number of rounds played. A** With each passing round, volunteers in both control and decoy treatments replace cooperation with defection, but the replacement pace is much faster in the decoy treatment. The average use of reward in the decoy treatment is constant and low. **B** Statistical analyses confirm these qualitative observations. Here, we fitted the data on cooperation and defection from the decoy treatment simultaneously, and calculated the frequency of reward using $x_R = 1 - x_C - x_D$. The benefit of such a fitting procedure was that we could readily see whether the intercept and the slope for, say, cooperation (Δb_C and Δa_C, respectively) were significantly different from the common intercept and slope, b and a. These two parameters also determined how the reward frequency changed with the number of rounds played. Accordingly, cooperation in the decoy treatment decreases $\approx \frac{-0.045}{-0.009} = 5$ times faster than in the control treatment, while the reward frequency "decreases" from $1 - 2 * b \approx 6.4\%$ at a pace $-a \approx 0$.

Supplementary discussion

Our results point to an intriguing, if somewhat speculative, role of cognitive biases in the evolution of human cooperation. Namely, recent research[8] shows that given the complex environmental conditions, as opposed to the (over)simplified laboratory ones, cognitive biases may be evolutionarily advantageous despite being responsible for seemingly irrational behaviour. Transitivity and regularity, for example, both of which closely relate to the independence of irrelevant alternatives (IIA) axiom of the rational choice theory, can be violated if decision rules are adapted to heterogeneous and autocorrelated environments[9,10]. In the context of human cooperation, therefore, it would seem that complex environments and the resulting cognitive biases accidentally[11] primed us to adopt more permissive and tolerant attitude towards prospective gift-bearers. Exploring such a possibility is surely worthwhile because increased permissiveness and tolerance may have opened the door just enough for cooperation to take root and generate benefits to early cooperators. These benefits may have transformed to a decisive evolutionary advantage over time, thus laying the foundations for the rise of modern cooperative societies.

Supplementary References

1. Dreber, A., Rand, D. G., Fudenberg, D. & Nowak, M. A. Winners don't punish. *Nature* **452**, 348–351 (2008).

2. Wu, J.-J. *et al.* Costly punishment does not always increase cooperation. *Proc. Natl. Acad. Sci. U.S.A.* **106**, 17448–17451 (2009).

3. Wang, Z. *et al.* Onymity promotes cooperation in social dilemma experiments. *Sci. Adv.* **3**, e1601444 (2017). URL `http://dx.doi.org/10.1126/sciadv.1601444`.

4. Fischbacher, U. z-Tree: Zurich toolbox for ready-made economic experiments. *Exper. Econ.* **10**, 171–178 (2007).

5. Wang, Z., Kokubo, S., Jusup, M. & Tanimoto, J. Universal scaling for the dilemma strength in evolutionary games. *Phys. Life Rev.* **14**, 1–30 (2015).

6. Tudge, S. J. & Brede, M. A tale of two theorems: Comment on "Universal scaling for the dilemma strength in evolutionary games" by Z. Wang et al. *Phys. Life Rev.* **14**, 49–51 (2015).

7. Taylor, C. & Nowak, M. A. Transforming the dilemma. *Evolution* **61**, 2281–2292 (2007).

8. Fawcett, T. W. *et al.* The evolution of decision rules in complex environments. *Trends Cogn. Sci.* **18**, 153–161 (2014).

9. Houston, A. I., McNamara, J. M. & Steer, M. D. Violations of transitivity under fitness maximization. *Biol. Lett.* **3**, 365–367 (2007).

10. Trimmer, P. C. Optimal behaviour can violate the principle of regularity. *Proc. R. Soc. B* **280**, 20130858 (2013). URL `http://dx.doi.org/10.1098/rspb.2013.0858`.

11. Gee, H. *The accidental species: Misunderstandings of human evolution* (University of Chicago Press, 2013).
